# Comparison of Complications, Metalwork Removal and Cost Between Locking and Tubular Plates for Lateral Malleolus Fractures Fixation

**DOI:** 10.7759/cureus.36181

**Published:** 2023-03-15

**Authors:** Rajesh Gopireddy, Ahmed T Hafez, Muhammad J Khan, Omar Javed, Islam Omar, Simon Clint

**Affiliations:** 1 Trauma and Orthopaedics, Gloucestershire Hospitals NHS Foundation Trust, Gloucester, GBR; 2 Surgery, Barts Health NHS Trust - Royal London Hospital, London, GBR; 3 Trauma and Orthopaedics, Gloucestershire Royal Hospital, Gloucester, GBR; 4 Surgery, Northern Health and Social Care Trust, Antrim, GBR

**Keywords:** orif, tubular plates, osteosynthesis, locking plates, ankle fractures

## Abstract

Introduction

Ankle fractures are common injuries in orthopaedic practice. Open reduction with internal fixation is the main line of management of displaced ankle fractures in fit patients. The study aims to analyse the complications, re-operation rate and cost difference between one-third tubular and locking plates which are the most frequently used constructs in lateral malleolus fractures.

Materials and methods

The total number of presented ankle fractures from April to August during the years 2015, 2017 and 2019 to our Tertiary Hospital in the United Kingdom were screened. Data including operative fixation, plate used, complication rates, the need for revision surgery and metalwork removal were collected from the hospital’s electronic Virtual Trauma Board. Patients who had less than one-year follow-up were excluded.

Results

A total of 174 patients were included which represents more than half of presented ankle fractures (56%) with a decline in the mean age of operated patients from 56.4 in 2015 to 46.2 in 2019. The majority of fixation used tubular plates (n=122) versus (n=52) for locking plates. Locking plate fixation doubled from 10 in 2015 to 23 in 2019. However, they only contributed to 27% of the total operated ankle fractures. Despite the initial higher complications and removal rates of locking plates in 2015 (P<0.042 and P<0.038 respectively), there was no significant difference in overall complications, revision rates, and metalwork removal between locking plates and tubular plates (p=0.084, FEp= 0.158 and p=0.096 respectively). There was an estimated extra cost of £15938.60 for the use of locking plates during the study timeline.

Conclusion

There was no significant difference in overall complications, revision surgery and metalwork removal between tubular and locking plates in treating lateral malleolus fractures despite the significantly higher cost of locking constructs. Further studies are needed to illustrate the trend and cost-effective analysis of the tubular and locking plates in treating ankle fractures.

## Introduction

Ankle fractures are common orthopaedic presentations in emergency departments with an approximate incidence of 180 per 100,000 persons per year [1,2]. It was reported to be the third most common fracture following wrist and hip fractures in the elderly population [3,4] and the fourth most common fracture in the adult population according to the Swedish fracture registry in 2013 [5]. Twisting injury is the mechanism of trauma in 69% of cases followed by a fall from height (10%) [6]. The epidemiology of fractures has changed over the past few decades, with initial reports of injury predominance in males [2,7]. However, recent studies point to a higher injury rate in females, especially during the post-menopausal period [6,8,9]. In 2016, the British Orthopaedic Association released guidelines [10] for ankle fracture management recommending the surgical fixation of displaced ankle fractures in fit patients less than 60 years and close contact casting for the elderly. Analysis of the trend of ankle fracture management in England over 10 years revealed that around 30% of the total ankle fractures underwent internal fixation with an increasing tendency towards surgical fixation in the elderly population [11].

Locking and tubular plates are the most commonly used implants among the different types available. Although the locking plates are preferred for highly comminuted fractures and in osteoporotic cases [12], tubular plates are quite popular because of the simplicity of fixation and the comparable functional outcomes and complications rate [13,14]. In 2016/2017, the cost of inpatient care for ankle fractures in England was estimated to be around 63 million annually [11] which represents a significant financial burden on the healthcare system in the United Kingdom. Our study aims to discuss the trend of using anatomical (locking) versus tubular plates, the complication rate, the need for revision surgery and the rate of metalwork removal in both constructs from 2015 to 2019, in addition to comparing the expenses of both constructs.

## Materials and methods

Study design

We conducted a retrospective comparative study using the electronic vault of the Virtual Trauma Board (VTB) for patients presented with ankle fractures from April to August in 2015, 2017 and 2019.

Setting

Trauma and Orthopaedics Department at a tertiary hospital in the United Kingdom. The Research Committee of Gloucestershire NHS Foundation Trust has granted approval No. SG200802 for this project.

Participants

We included all patients who underwent internal fixation of lateral malleolus of closed ankle fractures (Weber B and C) from April to August in 2015, 2017 and 2019 with a minimum of 12 months follow-up. We chose the same period every year in an attempt to get the same pattern of injuries. To assess the trend over time, data collection was done every other year starting from 2015, when our electronic medical records system began. The availability of plain radiographs and clinic letters was mandatory to obtain follow-up data. Open ankle fractures were not included in the study as they were not managed in our institution.

Variables examined

The primary outcomes were the complication rates, the incidence of revision surgery and the rate of metalwork removal. The secondary outcome was assessing the trend of using locking and tubular plates to fix ankle fractures over the study period. Additionally, we calculated the grade of the American Society of Anesthesiologists (ASA), associated comorbidities and age in each category. The total number of plates and screws was recorded for each case with their corresponding type (plates were either locking or one-third tubular plates, and screws were either locking or cortical).

Data source/bias

Data was collected from our electronic medical records. Operative notes and the imaging system were used to review the plate's type and the number of screws. General practitioner and clinic letters were studied to extract the different follow-up parameters and possible complications.

Study size

Our electronic database revealed 342 patients with ankle fractures presented during the time frame of the study, of which 191 patients only underwent surgical fixation. From April to August 2015, a total of 59 ankle fractures were managed surgically. During the same period of years 2017 and 2019, a total of 66 and 66 patients respectively underwent surgical treatment. Only 174 patients who had either locking or tubular plate fixation with follow-up of more than one year were included in the study. The approach used in our centre is the conventional lateral positioning of the plate.

Statistical analysis

Encounters are expressed in number and percentage. We used Microsoft Excel® to tabulate our data and calculate the percentage. Data were analysed using IBM (Statistical Package for the Social Sciences) SPSS software package version 20.0 (Armonk, NY: IBM Corp). The Kolmogorov-Smirnov test was used to verify the normality of the distribution of variables. Comparisons between groups for categorical variables were assessed using the Chi-square test (Fisher's Exact or Monte Carlo correction). Student t-test was used to compare two groups for normally distributed quantitative variables. The significance of the obtained results was judged at the 5% level.

## Results

A total of 342 patients presented to our hospital with ankle fractures from April to August in three years (2015, 2017 and 2019), of which 191 cases had internal fixation (56%). Only 174 fixations were included in the study with 122 patients treated with one-third tubular plates while only 52 had locking plates fixation. We excluded a total of 17 patients as they lost their follow-up less than one year following their operation. There was a steady increase in the use of locking plates from 19.6% in 2015 to 37.1% in 2019. Table 1 demonstrates the proportion of patients in both groups throughout the three years of study.

**Table 1:** Trend of plate selection over the years p1: p value for comparing 2015 and 2017 p2: p value for comparing 2015 and 2019 p3: p value for comparing 2017 and 2019 *: Statistically significant at p ≤0.05

	2015	2017	2019
Locking vs. Non-locking	(n = 51)	(n = 61)	(n = 62)
Anatomical (Locking)	10 (19.6%)	19 (31.1%)	23 (37.1%)
Tubular (Non-Locking)	41 (80.4%)	42 (68.9%)	39 (62.9%)
Sig. bet. grps.	p_1_=0.165, p_2_=0.042^*^, p_3_=0.487		

There were a few demographic differences between groups during the study period. In 2015, younger (≤60) patients received 80% of all locking plates used but only 41.5% of non-locking plates were used for the same age group (P=0.038) (Table 2).

**Table 2:** Comparison between the anatomical (locking) and tubular (non-locking) according to the demographic and outcome parameters in 2015 (n = 51) c2: Chi-square test; FE: Fisher exact; MC: Monte Carlo; ASA: American Society of Anesthesiologists p: p-value for comparing the studied groups *: Statistically significant at p ≤0.05

2015	Plate type		χ^2^	p
	Anatomical (Locking) (n = 10)	Tubular (Non-Locking) (n = 41)		
Demographics				
Age (years)				
≤60	8 (80%)	17 (41.5%)	4.777^*^	^FE^p=0.038^*^
>60	2 (20%)	24 (58.5%)		
Gender				
Male	3 (30%)	9 (22%)	0.289	^FE^p=0.682
Female	7 (70%)	32 (78%)		
ASA level				
Level I	6 (60%)	22 (22)	1.087	^MC^p=0.843
Level II	2 (20%)	12 (29.3%)		
Level III	2 (20%)	6 (14.6%)		
Level IV	0 (0%)	1 (2.4%)		
	8 (80%)	34 (82.9%)	0.047	^FE^p=1.000
≥ Level III	2 (20%)	7 (17.1%)		
Outcome Parameters				
Complications	5 (50%)	7 (17.1%)	4.844^*^	^FE^p=0.042^*^
Need for revision	0 (0%)	2 (4.9%)	0.508	^FE^p=1.000
Metalwork removed	4 (40%)	4 (9.8%)	5.560^*^	^FE^p=0.038^*^

In 2017, there was a significant difference in gender distribution between the two groups with female predominance in the locking plate group (P=0.033) as shown in (Table 3).

**Table 3:** Comparison between the anatomical (locking) and tubular (non-locking) according to the demographic and outcome parameters in 2017 (n = 61) c2: Chi-square test; FE: Fisher exact; MC: Monte Carlo; ASA: American Society of Anesthesiologists p: p-value for comparing the studied groups *: Statistically significant at p ≤0.05

2017	Plate type		χ^2^	p
	Anatomical (Locking) (n = 19)	Tubular (Non-Locking) (n = 42)		
Demographics				
Age (years)				
≤60	12 (63.2%)	30 (71.4%)	0.417	0.518
>60	7 (36.8%)	12 (28.6%)		
Gender				
Male	4 (21.1%)	21 (50%)	4.532^*^	0.033^*^
Female	15 (78.9%)	21 (50%)		
ASA level				
Level I	10 (52.6%)	28 (66.7%)	1.845	^MC^p=0.486
Level II	6 (31.6%)	7 (16.7%)		
Level III	3 (15.8%)	7 (16.7%)		
Level IV	0 (0%)	0 (0%)		
< Level III	16 (84.2%)	35 (83.3%)	0.007jn	^FE^p=1.000
≥ Level III	3 (15.8%)	7 (16.7%)		
Outcome Parameters				
Complications	7 (36.8%)	9 (21.4%)	1.606	^FE^p=0.224
Need for revision	2 (10.5%)	0 (0.0%)	4.571	^FE^p=0.093
Metalwork removed	5 (26.3%)	7 (16.7%)	0.771	^FE^p=0.489

There was no significant difference in any of the outcome parameters between the two groups. In 2019 (Table 4), there was no significant difference between the two groups in any studied parameters.

**Table 4:** Comparison between the anatomical (locking) and tubular (non-locking) according to the demographic and outcome parameters in 2019 (n = 62) c2: Chi-square test; FE: Fisher exact; MC: Monte Carlo; ASA: American Society of Anesthesiologists p: p-value for comparing the studied groups *: Statistically significant at p ≤0.05

2019	Plate type		χ^2^	p
	Anatomical (Locking) (n = 23)	Tubular (Non-Locking) (n = 39)		
Demographics				
Age (years)				
≤60	17 (73.9%)	34 (87.2%)	1.745	^FE^p=0.302
>60	6 (26.1%)	5 (12.8%)		
Gender				
Male	10 (43.5%)	9 (23.1%)	2.833	0.092
Female	13 (56.5%)	30 (76.9%)		
ASA level				
Level I	14 (60.9%)	31 (79.5%)	4.086	^MC^p=0.091
Level II	8 (34.8%)	5 (12.8%)		
Level III	1 (4.3%)	3 (7.7%)		
Level IV	0 (0%)	0 (0%)		
< Level III	22 (95.7%)	36 (92.3%)	0.268	^FE^p=1.000
≥ Level III	1 (4.3%)	3 (7.7%)		
Outcome Parameters				
Complications	4 (17.4%)	7 (17.9%)	0.003	^FE^p=1.000
Need for revision	1 (4.3%)	0 (0%)	1.723	^FE^p=0.371
Metalwork removed	2 (8.7%)	3 (7.7%)	0.020	^FE^p=1.000

More than two-thirds (67.8%) of all operated patients were ≤ 60 years old (mean 50.8 years), with the oldest patient aged 86. In locking plate fixation, 28.8% were more than 60 years compared to 33.6% in the tubular plate group with no significant difference (P=0.538). There were no significant differences in the other demographics, ASA score or comorbidities. See Table 5 comparing the anatomical and tubular plates groups according to the demographic and outcome parameters in the total sample.

**Table 5:** Comparison between the anatomical (locking) and tubular (non-locking) according to the demographic and outcome parameters in the total sample (n = 174) c2: Chi-square test; FE: Fisher exact; MC: Monte Carlo; ASA: American Society of Anesthesiologists p: p-value for comparing the studied groups *: Statistically significant at p ≤0.05

	Plate type		χ^2^	p
	Anatomical (Locking) (n = 52)	Tubular (Non-Locking) (n = 122)		
Demographics				
Age (years)				
≤60	37 (71.2%)	81 (66.4%)	0.379	0.538
>60	15 (28.8%)	41 (33.6%)		
Gender				
Male	17 (32.7%)	39 (32%)	0.009	0.925
Female	35 (67.3%)	83 (68%)		
ASA level				
Level I	30 (57.7%)	81 (66.4%)	2.866	^MC^p=0.419
Level II	16 (30.8%)	24 (19.7%)		
Level III	6 (11.5%)	16 (13.1%)		
Level IV	0 (0%)	1 (0.8%)		
< Level III	46 (88.5%)	105 (86.1%)	0.182	0.669
≥ Level III	6 (11.5%)	17 (13.9%)		
Comorbidities				
HTN	5 (9.6%)	20 (16.4%)	1.361	0.243
DM	2 (3.8%)	7 (5.7%)	0.266	^FE^p=0.726
Outcome Parameters				
Complications	16 (30.8%)	23 (18.9%)	2.977	0.084
Need for revision	3 (5.8%)	2 (1.6%)	2.228	^FE^p=0.158
Metalwork removed	11 (21.2%)	14 (11.5%)	2.776	0.096

The overall complication rate was higher with the locking plates (30.8%) compared to the tubular plates (18.9%) with no statistical significance (P=0.084). This is despite the initially significantly higher complication rate in locking plates (P=0.042) in 2015, with 40% of the locking plates removed compared to 9.8% in the tubular plate group (P=0.038). Throughout the study, the complications of locking plates were limited to prominent metalwork, tendon irritation, delayed wound healing, loose metalwork, improper reduction with talar shift of the ankle, wound infections and dehiscence. The patients with tubular plates had similar complications in addition to non-union, malunion and peroneal tendon irritation. Table 6 shows the different complications encountered in the two groups.

**Table 6:** Complications in each of the anatomical (locking) and tubular (non-locking) groups

Complication	Plate type	
	Anatomical (Locking)	Tubular (Non-Locking)
Infection and wound dehiscence	3	4
Delayed wound healing	3	3
Prominent screws and tendon irritation	1	1
Prominent metalwork	2	5
Painful scar and palpable implant	2	3
Malunion	0	1
Non-union	0	1
Loose metalwork	2	2
Unreduced fracture/residual diastasis	3	1
Peroneal tendon irritation	0	2

The cost of the plates was calculated according to the latest tariff. In each case, the plate and number of screws were identified. The mean cost of the locking plate was determined at £220.42, while the cost of a tubular plate was only £16.55. Similarly, 3.5mm locking screws costs £31.56 each, while non-locking screws cost £6.49. The total cost of plates and associated screws was calculated for each case. From 2015, the cost of locking plates and screws was more than double the cost of total locking metalwork (£4388.3 vs £2184.2) which increase significantly with the increased use of locking constructs to reach 4.9 times the cost of tubular metalwork (P<0.001) in 2019. It was estimated that the total cost of using locking constructs was £22477.70 and that we could have saved around £15938.60 if tubular plates and cortical screws had been used instead. Table 7 showed the analysis of the cost difference between the two study groups.

**Table 7:** Comparison between the anatomical (locking) and tubular (non-locking) according to cost SD: standard deviation; t: Student t-test p: p-value for comparing the studied groups *: Statistically significant at p ≤ 0.05

Cost	Plate type		t	p
	Anatomical (Locking)	Tubular (Non-Locking)		
2015 (n = 51)	(n = 10)	(n = 41)		
Mean ± SD	438.8 ± 18.2	53.3 ± 5.4	66.446^*^	<0.001^*^
Median (Min.-Max.)	441.3 (409.8-472.9)	55.5 (36-62)		
Sum	4388.3	2184.2	Saving = 2204.1	
2017 (n = 61)	(n = 19)	(n = 42)		
Mean ± SD	444.4 ± 31.8	56.9 ± 7.5	52.480^*^	<0.001^*^
Median (Min.-Max.)	441.3 (409.8-504.5)	55.5 (36-68.5)		
Sum	8443	2389	Saving = 6054	
2019 (n = 62)				
Mean ± SD	438.5 ± 40	51.7 ± 6	45.004^*^	<0.001^*^
Median (Min.-Max.)	441.3 (346.7-504.5)	49 (42.5-62)		
Sum	9646.4	1965.8	Saving = 7680.6	
Total sample (n = 172)				
Mean ± SD	440.7 ± 33.2	54 ± 6.7	82.390^*^	<0.001^*^
Median (Min.-Max.)	441.3 (346.7-504.5)	55.5 (36-68.5)		
Sum	22477.7	6539.1	Saving = 15938.6	

In this review, there were (58) uni-malleolar, (74) bi-malleolar and (40) tri-malleolar ankle fractures included. Weber B and C lateral malleolus fractures were included below (Table 8).

**Table 8:** Distribution of Weber classification of lateral malleolus fractures in the years of study

	2015		2017		2019	
Weber Classification	B	C	B	C	B	C
Anatomical (Locking)	9	1	12	7	17	6
Tubular (Non-Locking)	34	7	28	14	29	10
Total	43	8	40	21	46	16

## Discussion

More than two-thirds of our patients who were managed surgically were ≤ 60 years old. This is in keeping with the British Orthopaedic Association Standards for Trauma and Orthopaedics "BOASTs" guidelines released in 2016 [10] recommending surgical fixation in displaced ankle fractures in healthy, fit patients under 60 years old and close contact casting for elderly patients to minimise post-operative complications. However, many studies expected a rise of 25% in these ankle injuries in the elderly by 2050 due to increased life expectancy [2,4,15]. Juto et al. [16] reviewed 1756 cases of ankle fractures from 2009 to 2013 and found that one-third of the patients were 65 years or older. Despite that, most of the studies reported that the majority of patients presenting with ankle fractures remained under 60 years old [6,7] which was similar to our study findings with 68.7% of the patients under 60 years old.

Regarding gender, females represented approximately two-thirds of the study size (61.7%), which was consistent with recent studies reporting that the majority of ankle fractures occur in women, especially in the perimenopausal period [6,9].

Locking constructs have been gaining popularity among orthopaedic surgeons aiming to obtain adequate fixation in osteoporotic bone with poor quality and comminution [17]. Biomechanically, several studies demonstrated the superiority of locking constructs in providing higher torque failure rates when compared to conventional non-locking constructs. Davis et al. [18] tested the axial and torsional loading in 24 cadaveric specimens with four types of plates, which included non-locking and locking types of both one-third tubular and periarticular, and found that the non-locking plates had a greater tendency to fail with higher torque while locking plates had higher rotational stiffness, but there was no difference in axial loading. In another study, White et al. [19] tested type C Weber fractures treated with either a locking or non-locking construct in eight cadavers. They found no difference in torque to failure, stiffness, or axial or torsional values, which was also emphasised by Eckel et al. [20], in testing Weber B fractures.

Our study showed an increased inclination towards using locking plates for the internal fixation of ankle fractures. This was accompanied by a steady decline in the associated complication rates from 50% in 2015 to a level comparable to the tubular plates in 2019, with 17.4% for the former and 17.9% for the latter. The evolution of locking plate utilisation was associated with a parallel decline in metalwork removal from 40% in 2015 to 8.7% in 2019. However, the overall complications associated with locking plates were significantly higher than the tubular plates.

During the initial use of the locking plate systems in early 2015, there were higher complications and revision rates compared to conventional tubular plates. This was in accordance with a warning issued by Schepers et al. [21], in 2011 following his findings of a significant rise in wound complications following locking plate fixation in distal fibular fractures (17.5% in locking plates vs 5.5% in conventional ones). Wound complications might be attributed to the thickness of the locking plates which can compromise the wound vascularity as it places the skin under tension [22]. An additional reason behind complications in the early stages of locking plate utilisation is the lack of knowledge of the principles of this relatively new technology at that time, leading to multiple failures and a longer learning curve for the surgical technique [23].

With the growing familiarity with the biomechanics relevant to the locking constructs, the complication rates fell significantly down to levels comparable to that of the tubular plates in the following years of our study with no significant difference in overall complication rate, metalwork removal or need of revision between locking and tubular plates in the whole sample collectively. This appears to be consistent with several studies, including a retrospective analysis by Lynde et al. [24], of 216 elderly patients who sustained ankle fractures with no differences in complication rate, failure of fixation or hardware removal between conventional and locking plates. Moreover, another retrospective analysis by Lyle et al. [25] revealed no significant difference in complication rate and re-operation rate (p=0.291) between three groups of patients treated with locking, semi-tubular or dynamic compression plates. The latter results were also demonstrated by another comparative study by Moriarity et al. [26], which compared the overall complication rate and wound complication rates of 160 patients and found no significant differences (P=0.76 and P=1.00, respectively). Another study that included 52 patients randomised to either a locking or neutralisation plate for ankle fixation showed no difference in time to fracture union (radiological and clinical), short-form assessment-36 and complication rates between both groups [13].

Further studies focused on ankle fracture fixations in the elderly population; Shih et al. [27] reviewed patients above 50 years old with ankle fractures that underwent locking (particularly periarticular) or tubular plating. They concluded that there were no significant differences in terms of overall and wound complications. However, the radiological outcomes, including more than 2mm fibular shortening and distal fibular screw loosening, in addition to one-year Foot and Ankle Outcome Score (FAOS) and Visual Analogue Scale (VAS), were significantly better in the locking group. Additionally, H-Perez et al. [28] found an earlier partial weight-bearing in elderly osteoporotic patients managed with locking plates compared to conventional ones. The complications were associated with ageing and diabetes as per Lynde et al. [24] but without statistical significance. In this study, we had 39 cases with complications, 18 of which were associated with comorbidities (46%) with no significant difference regarding comorbidities in both study groups.

The financial burden of locking plates has been discussed in several studies demonstrating the massive impact of the locking system’s cost compared to the conventional ones such as one-third of tubular plates. Approximately 60,000 locking plates are used each year in the United States, with an estimated cost of an extra $800 for the locking compared to the tubular plates. A different study from the USA suggested that the saving would reach $50 million annually if tubular plates replaced the costly locking plates for ankle fracture fixation [29]. Another economic analysis revealed that the cost of the anatomical plate is approximately $746.97. In contrast, the tubular plate would cost only $90.86, resulting in a significant saving of approximately $38 million if one-third of tubular plates replaced the locking plates when clinically justified [30]. Our analysis showed that there is a continuous rise in the cost of ankle fracture fixation in view of the increasing use of locking plates with a mean value of £440.70 ± 33.2 for the locking constructs (including plate and screws), which is eight times more costly than the tubular plates at an average cost of £54 ± 6.7 (p<0.001). It was estimated that we could have saved a total of £15938.6 in a single hospital during the three study periods, despite fewer patients being managed by locking plates. However, this could be justified by the clinical indication to utilise locking plates in older patients with osteoporotic bones to avoid construct failure and the costs of readmissions and re-interventions. Further studies are needed to demonstrate the cost-effectiveness of different plates in lateral malleolus fixation indifferent patients’ groups. Hence this could be used on a selective basis.

Limitations of the study

The indication and choice of plate used for fixation is the surgeon’s own preference and depend on the clinical need. The study is limited in terms of full cost-effective analysis of the type of plate fixation as estimated time to return to work, the total number of post-operative follow-ups, cost of rehabilitation and physiotherapy sessions.

## Conclusions

There is an increasing trend among orthopaedic surgeons towards the fixation of ankle fractures with locking constructs. Despite the significantly higher cost of locking plates, tubular and locking plates had similar complication rates, metalwork removal and revision surgeries. The initial higher complication rates in the locking plate group could be related to the learning curve of the newly introduced implants. Further studies are needed to illustrate the trend and cost-effective analysis of lateral malleolus fixation in ankle fractures.
